# Efficacy of active and passive evidence-based practice training for postgraduate medical residents: a non-randomized controlled trial

**DOI:** 10.1186/s13104-021-05732-3

**Published:** 2021-08-19

**Authors:** Hassan Goodarzi, Ehsan Teymourzadeh, Siyavash Rahimi, Taha Nasiri

**Affiliations:** 1 Emergency Department, Trauma Research Center, Baqiyatallah University of Medical Sciences, Tehran, Iran; 2 Health Management Research Center, Baqiyatallah University of Medical Sciences, Tehran, Iran; 3 Department of Health Services Management, Faculty of Health, Baqiyatallah University of Medical Sciences, Tehran, Iran

**Keywords:** Postgraduate medical education, Evidence-based practice, Medical education

## Abstract

**Objective:**

This study examined the effects of two evidence-based practice (EBP) educational programs for postgraduate medical residents on their attitude, behavior, knowledge, outcome, and competencies in EBP.

**Results:**

Forty-five and thirty-five medical residents were recruited in the active and passive educational intervention groups, respectively. Among those, 39 and 30 participants were included in the final analysis. The participants of the active group received 12 h of EBP-structured presentation. The passive educational group received EBP education through their daily rounds, evidence-based journal clubs, and morning reports. Participants were evaluated with EBP-KABQ and ACE tools questionnaires. The active and passive intervention groups were not significantly different from each other at the baseline in the EBP-KABQ questionnaire and ACE tools score (p > 0.05). However, most questions in the EBP-KABQ questionnaire were significantly different from the pre-intervention measurement and the passive intervention group after the educational intervention. Educational intervention in both groups led to a significant difference in ACE tools score between groups (8.86 ± 2.62 vs. 7.31 ± 2.92, p = 0.029, in the active and passive groups, respectively). Paired t-test analysis revealed that our intervention led to a significant increase in ACE tool scores in both groups (p < 0.000, in both groups).

**Supplementary Information:**

The online version contains supplementary material available at 10.1186/s13104-021-05732-3.

## Introduction

Evidence-based practice (EBP) is defined as incorporating the best available body of evidence with clinical judgment in the framework of patient preferences, values, and characteristics [1, 2], which is known to improve the quality of care in medicine [3]. Therefore, knowledge of the principles of EBP and skills to apply the steps of the EBP implementation process are essential competencies for all practicing healthcare professionals [4]. Blooming in the information technologies, clinical practice, and research methodology in recent years have made the implementation of evidence-based decision-making more desirable and feasible for medical practice [5]. Despite the fact that EBP has been shown to improve the quality of patient care, there has been a slow improvement in teaching EBP and incorporating it into medical school's curriculum [6, 7]. Therefore, many practicing healthcare professionals lack the skills and experiences to systematically transfer EBPs into their routine care [4]. Moreover, medical professions in Iran still habitually overvalue personal information resources or empirical knowledge, rather than research outcomes, to make decisions or overcome clinical settings uncertainties. In the most recent domestic research, the actual application of the EBP in the field of practice is 15.8% [8].

EBP is primarily trained as seminars or short courses. A systematic review by Coomarasamy and Khan revealed that stand-alone teaching of EBP only improved trainees’ knowledge, but not skills, attitudes, or behavior in EBP. They suggested that EBP education should be integrated into clinical practice rather than stand-alone classroom education [9]. Ilic and Maloney’s most recent systematic review showed that the learner’s outcomes across various teaching modalities such as workshops, didactic lectures, directed and self-directed learning, small group work, online and computer-assisted learning, and multidisciplinary and discipline-specific problem-based learning group were similar [10]. However, only one study was performed on postgraduate students, and the passive teaching method was not evaluated [10, 11]. Therefore, the efficacy of passive teaching modality is remained to be elucidated.

Recently, the adaptation of EBP principles into medical education has gain attention in Iran, and it is implemented into undergraduate and postgraduate training curriculums [6]. Teaching EBP to academic teachers, using evidence-based-oriented textbooks as a reference, evidence-based journal clubs, and morning reports are among the measures taken to improve postgraduate students' competencies in EBP [6]. Although this seems to be a good starting point for postgraduate training, the value of course-based teaching of EBP has not been emphasized in the current curriculum. Furthermore, unlike undergraduate students, interventions on postgraduate students do not lead to robust improvements; therefore, multiple educational modalities should be implemented to improve postgraduate training outcomes [9, 12].

Thus, based on this background, in this study, we carried out an EBP course-based program for postgraduate year 1 (PGY-1) and medical residents and evaluated their attitude, behavior, knowledge, and outcome in EBP. Furthermore, we compared the results to our PGY-2 residents who were subjected to passive EBP education for a year.

This study was designed to compare the attitude, behavior, knowledge, outcome, and competencies of postgraduate trainees in two evidence-based practice (EBP) educational programs.

## Main text

### Methods

#### Trial design

In this non-randomized controlled trial, PGY-1 and PGY-2 medical residents were studied at Baqiyatallah Hospital of Tehran, Iran. All medical residents were presented with a briefing about the EBP assessment thereafter, they filled the questionnaires. The participants allocated to the active group received a weekly 2 h EBP-structured presentation covering EBP approaches to patient care experiences offered by the EBM faculty team of the Baqiyatallah University of Medical Sciences. Participants of the passive education group were assigned to our new EBP-oriented curriculum for a year. Teachers and participants were all informed about the courses they were going to attend. Therefore, blinding and allocation concealment were not possible in the present study. Nevertheless, the intention of the study had not been disclosed to the participants. Written informed consent was obtained from each participant. All procedures were approved by the ethics committee of Baqiyatallah University of Medical Sciences (approved no: IR.BMSU.BAQ.REC.1398.020). Participants who did not consent to participate in the study or did not attend at least 50% of classes were excluded from the study.

#### Educational intervention

The educational intervention of the EBP course was developed by qualified EBP professors of Baqiyatallah University of Medical Sciences. This course was intended to provide an interactive forum for participants to improve the clinical implementation of EBM. The primary outcome of this study was knowledge, attitudes, outcome/decision, and behavior, which was measured by using the previously validated evidence-based practice knowledge, attitude, behavior questionnaire (EBP-KABQ) [13]. Furthermore, competency of participants in EBP was measured using Assessing Competency in EBM tool (ACE tool). Due to the busy schedule of medical residents and the COVID-19 pandemic, presentations were given in-class and online for only two hours per week. Therefore, participants could attend either class as they preferred. Classes were according to the COVID-19 precaution protocols. Because of the current time pandemic and involving all medical residents, most examples, articles, and presentations were based on COVID-19. In total, participants in the active group received a 12-h EBP course. The training course is outlined in Additional file 1: Table S1. The introduction session provided an overview of EBP in brief. The following four sessions were created based on the 5-step model of EBP principles, consist of the development of clinical question and search strategy, a systematic literature search of medical databases, critical appraisal, and evidence synthesis, apply the evidence to the relevant case scenario. Each session was devoted to a single study type (observational, diagnostic, therapeutic, systematic review and meta-analysis studies). The final session (sixth session) presented three actual clinical examples of COVID-19-related dilemmas to demonstrate the real-time application of EBP skills. Also, trainees were familiarized with the evidence-based medicine self-evaluation (self-reflection) Toolbox. The EBM faculty teaching team consists of two professors and one novice teacher. Each session was taught by multiple teachers. Also, participants were allowed to ask questions and faculty members were responsive during the training period. The passive educational group received EBP education through their daily rounds, evidence-based journal clubs, and morning reports.

#### Evaluation instruments

##### Assessment of knowledge, attitude, outcome and behavior in participants

Knowledge, behavior, outcome or decision, and attitude were measured before and after EBP training using the EBP-KABQ tool. Assessment questionnaires consisted of 33 questions answered using the Likert scale [13]. In the knowledge and attitude section, higher scores indicate better knowledge and positive attitude, respectively. For EBP behavior, and outcomes lower scores indicate a lower frequency of using EBP and unfavorable patient outcomes and poor clinical evidence-based decision making, respectively.

#### Assessment of competency in EBP

Although the EBP-KABQ questionnaire has been developed to measure users' knowledge, behavior, and attitude, it does not examine the skills and competency of individuals in evidence-based medicine (EBM). Recently, The Assessing ACE tool has been developed by Ilict et al. [14]. This questionnaire presents a brief clinical scenario from which a clinical question is developed. Users are then presented with a search strategy and a hypothetical article extract. Users then work through 15 questions (answering yes or no), with each question representing a step in EBM. Items 1–11 assess knowledge and skills relevant to EBM, whilst items 12–15 assess attitudes relevant to EBM implementation in clinical practice. All questions are designed as yes or no answers. Each correct answer is awarded by one score with no penalty for wrong answers. The tool has been found to be a reliable and valid instrument to assess medical trainees’ competency in EBM [14]. This questionnaire was presented in the English language to our participants.

### Statistical analysis

Statistical analyses were performed using the SPSS software version 25.0 for Windows (SPSS Inc., Chicago, IL, USA). The Levene and Kolmogorov–Smirnov tests were used to examine the equality of variances and distribution of variables, respectively. In case of a normal distribution, an independent t-test was applied to compare mean values of quantitative variables, otherwise, the Mann–Whitney U test was used. The qualitative and quantitiave variables are presented as numbers (proportions), and mean ± standardized deviations (SDs). Differences in categorized variables was analyzed by Chi-square test. Before and after analysis was performed using paired t-test. All statistical tests were 2-tailed, and p < 0.05 was considered statistically significant.

### Results

#### General characteristics of participants

Forty-five medical residents were recruited for the active group, 39 participants attended more than 50% of classes. In the passive group, 30 out of 35 medical residents responded to our questionnaires. 26 (66.6%) participants in the active group and 20 (66.6%) participants in the passive group were males (p > 0.05). Participants in the active group were significantly younger than the passive group (28.18 ± 2.15 vs. 31.23 ± 3.22, p < 0.0001).

#### Knowledge, behavior, outcome or decision, and attitude of participants

Prior to initiation of classes, participants were instructed to answer the EBP-KABQ and ACE tool questionnaire. A month after the intervention, participants in the active group were asked to answer questionnaires once again. A year after passive intervention, the participants in the passive group were asked to answer the questionnaire once again. The result of the EBP-KABQ questionnaire is shown in Additional file 2: Table S2. Post-intervention results and comparison with pre-intervention and the passive groups are shown in Additional file 2: Table S2. The active and passive groups were not significantly different from each other at the baseline (p > 0.05). However, after the educational intervention, most questions were significantly different from the pre-intervention measurement and the passive group (see Additional file 2: Table S3).

#### EBP competency

The competency of participants in performing EBP was assessed using the ACE tools questionnaire. Before the educational intervention, both groups had similar scores (4.14 ± 1.72 in the active group vs. 4.79 ± 0.94 in the passive group, p > 0.05). However, educational intervention in both groups led to a significant difference between groups (8.86 ± 2.62 vs. 7.31 ± 2.92, p = 0.029, in the active and passive group, respectively). Paired t-test analysis revealed that our intervention has lead to a significant increase in ACE tool scores in both groups (p < 0.000, in both groups).

### Discussion

In the present study, we evaluated PGY-1 and PGY2 medical residents’ attitude, knowledge, behavior, and decision regarding EBP before and after the educational intervention. Also, the ACE tool was employed to assess the competency of individuals in EBP.

Although both educational interventions have been shown to be effective, the results of this study suggest that an EBP course is superior to exposure of postgraduate trainees to passive EBP practice. The participants in the EBP course showed significantly higher scores in knowledge, attitude, decision or outcome, and behavior compared to the passive group. This difference was more pronounced in the decision and attitude domain. We also employed the most recent and comprehensive tool for the evaluation of EBP competency. The ACE tool results showed that the EBP course results in higher scores compared to passive teaching of EBP. The result of previous studies regarding the efficacy of passive and active teaching of EBM is conflicting. Kumaravel et al. have performed a study on undergraduate medical students. They found that active teaching leads to higher educational prescription scores, while there was no significant difference in performances in the ACE tool or the summative assessments [15]. On the other hand, Draaisma et al. in a cross-sectional study, found that teaching EBM by deliberate usage was superior to standard EBM workshops because students viewed EBM as more useful and were more likely to use it in decision making than the other group [16]. The observed difference can be due to the long-term active deliberate training of postgraduate medical students (four years of training). Also, one group of individuals were clinical Ph.D. research program students.

Unlike the systematic review, which did not find any differences in learner outcomes across various teaching methods, our study has shown that passive and active teaching can result in different outcomes in the competency, attitude, knowledge, behavior, and decision of postgraduate medical residents regarding EBP [10]. The observed discrepancy can be due to the absence of any passive teaching method in the systematic review. It is suggested that passive teaching reduces the need for resources, while delivers the same quality [11]. However, evidence regarding EBP education is scarce, and additional research is needed. To the best of our knowledge, this is the first study that has compared the efficacy of passive versus active methods of EBP education in postgraduate students, which is evaluated by ACE tools and EBP-KABQ.

This study contributes to the current knowledge of passive EBP teaching in postgraduate trainees. Our study results suggest that an EBP course is better than one year of passive EBP exposure. Therefore, based on our current curriculum, stand-alone passive EBP exposure does not seem efficient, and a more blended approach should be used. Future studies should aim to assess the efficacy of blended approach of active teaching with continuation of education with passive teaching.

### Limitations

This study was a pragmatic trial; therefore, randomization or blinding individuals to the interventions was impossible. Due to the current curricular changes, passive teaching of EBP is implemented on all medical residents; therefore, all students, including the active group, could be exposed to this method. We chose newly introduced residents (PGY-1) unexposed to passive intervention and implemented the active intervention in a short time, and then we evaluated them after a month to minimize the effect of passive educational exposure. On the other hand, passive intervention takes time. Thus, we had to continue our passive education through the year and evaluate them after a year. The passive teaching method relies on student and teacher engagement in the EBP-oriented routine education; therefore, either party's disengagement will lead to poor outcomes and biased results.

## Supplementary Information

**Additional file 1**: EBP Educational program for postgraduate medical residents (active education group).

**Additional file 2**: Description: The EBP-KABQ questionnaire results in two groups before and after the intervention.

## Data Availability

All data generated or analyzed during this study are included in this published article and its Additional files.

## Abbreviations

EBP: Evidence-based practice
EBM: Evidence-based medicine
PGY: Postgraduate year
EBP-KABQ: Evidence-based practice knowledge, attitude, and behavior questionnaire
ACE tool: Assessing competency in EBM tool
SD: Standard deviation
