# Supplementary material for: Efficacy of active and passive evidence-based practice training for postgraduate medical residents: a non-randomized controlled trial
Source: BMC Res Notes. 2021 Aug 19;14:317. doi: 10.1186/s13104-021-05732-3 (PMC8374403; doi:10.1186/s13104-021-05732-3)
Supplement: Supplementary file 1 — Additional file 1: EBP Educational program for postgraduate medical residents (active education group). [file 13104_2021_5732_MOESM1_ESM.docx]

| **Table S1.** EBP Education Program for Postgraduate medical residents. | | | |
| --- | --- | --- | --- |
| Session | EBP steps | Content | Duration (hours) |
| 1 | - development of clinical question and search strategy - a systematic literature search of medical databases | - Importance of EBP - Hierarchy of evidence - Steps of EBP - Level of Evidence - Technologies for EBP application - Asking clinical question - PICO formulation - Database for searching evidence - Developing search strategies - Using search commands - Case-based exercises | 2 |
| 2 | - Critical appraisal of randomized controlled trials - Evidence synthesis and applying of randomized controlled trials | - Basic component of critical appraisal - Critical appraisal of randomized controlled trials - Case-based exercise | 2 |
| 3 | - Critical appraisal of observational studies - Evidence synthesis and applying of observational studies | - Critical appraisal of observational studies - Case-based exercise | 2 |
| 4 | - Critical appraisal of diagnostic studies - Evidence synthesis and applying of diagnostic studies | - Critical appraisal of diagnostic studies - Case-based exercise | 2 |
| 5 | - Critical appraisal of systematic reviews and meta-analysis - Evidence synthesis and applying of systematic reviews and meta-analysis | - Critical appraisal of clinical practice guidelines and systematic-review and meta-analysis studies - Case-based exercise - Implementation of recommendations - Evaluation of EBP process and outcomes | 2 |
| 6 | - Performance evaluation - Rehearsal | - Evidence-Based Medicine self-evaluation toolbox - Case-based exercises for all types of clinical research | 2 |
