# Supplementary material for: Efficacy of active and passive evidence-based practice training for postgraduate medical residents: a non-randomized controlled trial
Source: BMC Res Notes. 2021 Aug 19;14:317. doi: 10.1186/s13104-021-05732-3 (PMC8374403; doi:10.1186/s13104-021-05732-3)
Supplement: Supplementary file 2 — Additional file 2: Description: The EBP-KABQ questionnaire results in two groups before and after the intervention. [file 13104_2021_5732_MOESM2_ESM.docx]

| **Table S2**. EBP-KABQ questionnaire score in groups. | | |
| --- | --- | --- |
| **Question** | **Passive group (n= 30)** | **Active group (n= 39)** |
| **Domain of Knowledge** | | |
| 1. I am confident in my ability to use evidence-based practice. | 4.33 ± 1.58 | 4.54 ± 1.54 |
| 2. Using evidence-based practice increases the certainty that the proposed treatment is effective | 4.4 ± 1.25 | 4.33 ± 1.77 |
| 3. Research using clinical trials is generally more reliable than research using the observational method. | 3.2 ± 1.32 | 3.79 ± 1.84 |
| 4.It is important for me to search bibliographic databases to be an effective clinician. | 4.93 ± 0.94 | 5.05 ± 1.59 |
| 5. It is important for me to critically appraise research papers to be an effective clinician. | 4.53 ± 1.19 | 4.44 ± 1.19 |
| 6. Evidence and patients are equally important to making clinical decisions. | 4.53 ± 1.68 | 4.36 ± 1.95 |
| 7. What % of your patient decisions are based on evidence from clinical research? | 46.83 ± 21.23 | 50.9 ± 25.07 |
| 8. How frequently do you have questions about managing your patients that might require research evidence to answer (times per week on average)? | 14.83 ± 9.11 | 10.79 ± 8.24 |
| **Domain of (EBP) behavior** | | |
| 9. How frequently do you access clinical research evidence *in general*? | 3.1 ± 1.03 | 2.72 ± 1.12 |
| 10. How frequently do you access clinical research evidence *from a textbook*? | 2.63 ± 1.03 | 2.97 ± 1.13 |
| 11. How frequently do you access clinical research evidence *from original research papers*? | 2.57 ± 1.10 | 2.69 ± 0.97 |
| 12. How frequently do you access clinical research evidence *from the Cochrane database*? | 2.23 ± 0.97 | 2.2 ± 1.28 |
| 13. How frequently do you access clinical research evidence *from secondary sources such as ACP Journal Club, the journal Evidence-Based Medicine, POEMs (Patient-oriented evidence that matters) or CATs (Critically appraised topics)*? | 1.63 ± 0.61 | 1.41 ± 0.64 |
| 14. On average, how often do you now look up evidence immediately before, or during patient treatment visit? (hours per week) | 3.17 ± 1.82 | 2.67 ± 1.91 |
| 15. How many hours per week do you spend looking up evidence? | 2.47 ± 1.59 | 2.77 ± 1.31 |
| 16. How many hours per week do you spend reading new research evidence? | 2.03 ± 0.99 | 2.13 ± 1.13 |
| **Domain of (EBP) decisions and outcomes** | | |
| 17. How much has the use of evidence-based practice affected your clinical decisions? | 3.4 ± 1.04 | 3.74 ± 1.09 |
| 18. How much has the use of evidence-based practice affected your patient outcomes? | 4.4 ± 1.5 | 4 ± 1.28 |
| 19. How often does new research evidence result in a change in your practice? | 3.97 ± 0.99 | 4.33 ± 1.32 |
| **Domain of attitudes (towards EBP)** | | |
| 20. How much confidence do you have in your clinical decision-making | 3.4 ± 1.04 | 3.71 ± 1.19 |
| 21. Evidence-based practice is clinical performance guide which ignores clinical experience. | 4.9 ± 1.15 | 4.77 ± 1.06 |
| 22. It is easy to find the research. | 3.57 ± 1.3 | 2.97 ± 1.42 |
| 23. Evidence-based practice takes too much time. | 4.9 ± 1.27 | 5.44 ± 1.57 |
| 24. Evidence-based practice ignores the “art” of clinical practice. | 5.27 ± 1.23 | 5.59 ± 0.88 |
| 25. Previous clinical experience is more important than research findings in choosing the best treatment available for a patient. | 4.9 ± 0.8 | 5.26 ± 1.04 |
| 26. Evidence-based practice should be an integral part of clinical practice. | 4.33 ± 1.06 | 4.59 ± 1.02 |
| 27. From my personal observation and experience, evidence-based practice is being used currently by my colleagues. | 2.33 ± 1.12 | 2.64 ± 1.2 |
| 28. I use evidence-based practice because it improves patient outcomes. | 5.4 ± 1.07 | 5.26 ± 1.04 |
| 29. I use evidence-based practice because I believe in it. | 5.13 ± 1.78 | 5.49 ± 1.29 |
| 30. I use evidence-based practice because my colleagues do. | 2.87 ± 1.2 | 3.23 ± 1.13 |
| 31. I don’t use evidence-based practice because I don’t have time. | 5.2 ± 0.71 | 5.13 ± 1.42 |
| 32. I don’t use evidence-based practice because it is difficult to change. | 2.73 ± 0.69 | 2.41 ± 1.23 |
| 33. I don’t use evidence-based practice for another reason (specify): | 3.2 ± 1.3 | 3.18 ± 1.05 |
| Data are expressed as Mean ± SD | | |

| **Table S3**. EBP-KABQ questionnaire score in interventional group after educational intervention. | | |  |
| --- | --- | --- | --- |
| **Question** | **Active group (n= 39)** | **Passive group (n= 30)** |  |
| **Domain of Knowledge** | | |  |
| 1. I am confident in my ability to use evidence-based practice. | 5.23 ± 1.04^b^ | 4.93 ± 1.34 ^b^ |  |
| 2. Using evidence-based practice increases the certainty that the proposed treatment is effective | 5.72 ± 0.97 ^b^ | 5.13 ± 1.43 ^b^ |  |
| 3. Research using clinical trials is generally more reliable than research using the observational method. | 1.9 ± 1.16^a^ | 2.7 ± 1.51 ^b^ |  |
| 4.It is important for me to search bibliographic databases to be an effective clinician. | 6.2 ± 0.77 ^a,b^ | 5.53 ± 1.1 ^b^ |  |
| 5. It is important for me to critically appraise research papers to be an effective clinician. | 6.18 ± 0.76 ^a^ | 4.93 ± 1.11 |  |
| 6. Evidence and patients are equally important to making clinical decisions. | 6.2 ± 0.69 ^a^ | 4.9 ± 1.63 |  |
| 7. What % of your patient decisions are based on evidence from clinical research? | 68.33 ± 14.79 ^a,b^ | 53.17 ± 19.98 ^b^ |  |
| 8. How frequently do you have questions about managing your patients that might require research evidence to answer (times per week on average)? | 10.79 ± 2.37 ^b^ | 15.4 ± 7.48 |  |
| **Domain of (EBP) behavior** | | |  |
| 9. How frequently do you access clinical research evidence *in general*? | 4 ± 0.76 ^b^ | 3.6 ± 0.97 ^b^ |  |
| 10. How frequently do you access clinical research evidence *from a textbook*? | 3.36 ± 0.93 ^a,b^ | 2.63 ± 0.96 ^b^ |  |
| 11. How frequently do you access clinical research evidence *from original research papers*? | 3.95 ± 0.79 ^b^ | 3.53 ± 1.04 ^b^ |  |
| 12. How frequently do you access clinical research evidence *from the Cochrane database*? | 3.54 ± 1.02 | 3.3 ± 0.952 ^b^ |  |
| 13. How frequently do you access clinical research evidence *from secondary sources such as ACP Journal Club, the journal Evidence-Based Medicine, POEMs (Patient-oriented evidence that matters) or CATs (Critically appraised topics)*? | 3.56 ± .99 ^a,b^ | 2.43 ± 0.935 ^b^ |  |
| 14. On average, how often do you now look up evidence immediately before, or during patient treatment visit? (hours per week) | 3.59 ± 1.65 | 3.6 ± 1.79 |  |
| 15. How many hours per week do you spend looking up evidence? | 6.05 ± 3.35 ^a^ | 2.9 ± 1.18 |  |
| 16. How many hours per week do you spend reading new research evidence? | 3.87 ± 1.69 ^a^ | 3.13 ± 0.97 ^b^ |  |
| **Domain of (EBP) decisions and outcomes** | | |  |
| 17. How much has the use of evidence-based practice affected your clinical decisions? | 5.1 ± 0.79 ^a,b^ | 4.03 ± 1.06 ^b^ |  |
| 18. How much has the use of evidence-based practice affected your patient outcomes? | 5.33 ± 0.66 ^a,b^ | 4.93 ± 0.91 ^b^ |  |
| 19. How often does new research evidence result in a change in your practice? | 5 ± 0.86 ^a,b^ | 4.57 ± 0.9 ^b^ |  |
| **Domain of attitudes (towards EBP)** | | |  |
| 20. How much confidence do you have in your clinical decision-making | 4.43 ± 0.64 ^a,b^ | 4 ± 0.79 ^b^ |  |
| 21. Evidence-based practice is clinical performance guide which ignores clinical experience. | 2.38 ± 1.04 ^a^ | 3.57 ± 1.3 ^b^ |  |
| 22. It is easy to find the research. | 5.2 ± 0.8 ^b^ | 4.73 ± 1.17 ^b^ |  |
| 23. Evidence-based practice takes too much time. | 3.31 ± 1.43 | 3.73 ± 1.55 ^b^ |  |
| 24. Evidence-based practice ignores the “art” of clinical practice. | 2.26 ± 1.09 ^a^ | 3.8 ± 1.45 ^b^ |  |
| 25. Previous clinical experience is more important than research findings in choosing the best treatment available for a patient. | 2.2 ± 1.1 ^a^ | 3.66 ± 1.15 ^b^ |  |
| 26. Evidence-based practice should be an integral part of clinical practice. | 6.72 ± 0.51 ^a^ | 4.83 ± 1.02 ^b^ |  |
| 27. From my personal observation and experience, evidence-based practice is being used currently by my colleagues. | 3.46 ± 1.6 ^a,b^ | 2.5 ± 0.9 |  |
| 28. I use evidence-based practice because it improves patient outcomes. | 6.49 ± 0.76 ^a^ | 5.73 ± 1.01 ^b^ |  |
| 29. I use evidence-based practice because I believe in it. | 6.26 ± 0.91 ^a,b^ | 5.43 ± 1.41 |  |
| 30. I use evidence-based practice because my colleagues do. | 1.95 ± 0.92 ^a,b^ | 4.33 ± 1.37 ^b^ |  |
| 31. I don’t use evidence-based practice because I don’t have time. | 3.15 ± 1.69 ^b^ | 3.7 ± 1.7 ^b^ |  |
| 32. I don’t use evidence-based practice because it is difficult to change. | 1.61 ± 0.88 ^a,b^ | 2.13 ± 1.07 ^b^ |  |
| 33. I don’t use evidence-based practice for another reason (specify): | 3.92 ± 0.48 | 4 ^b^ |  |
| Data are expressed as Mean ± SD  a, p<0.05 vs. control group.  b, p<0.05 vs. previous measurement. | | |  |
